# Lithium in Drinking Water and Thyroid Function

**DOI:** 10.1289/ehp.1002678

**Published:** 2011-01-20

**Authors:** Karin Broberg, Gabriela Concha, Karin Engström, Magnus Lindvall, Margareta Grandér, Marie Vahter

**Affiliations:** 1 Division of Occupational and Environmental Medicine, Lund University, Lund, Sweden; 2 Division of Toxicology, Swedish National Food Administration, Uppsala, Sweden; 3 Department of Child and Youth Psychiatry, Lund University, Lund, Sweden; 4 Institute of Environmental Medicine, Karolinska Institutet, Stockholm, Sweden

**Keywords:** bipolar disorder, iodine, lithium, selenium, thyroid-stimulating hormone, thyroxine

## Abstract

**Background:**

High concentrations of lithium in drinking water were previously discovered in the Argentinean Andes Mountains. Lithium is used worldwide for treatment of bipolar disorder and treatment-resistant depression. One known side effect is altered thyroid function.

**Objectives:**

We assessed associations between exposure to lithium from drinking water and other environmental sources and thyroid function.

**Methods:**

Women (*n* = 202) were recruited in four Andean villages in northern Argentina. Lithium exposure was assessed based on concentrations in spot urine samples, measured by inductively coupled plasma mass spectrometry. Thyroid function was evaluated by plasma free thyroxine (T_4_) and pituitary gland thyroid-stimulating hormone (TSH), analyzed by routine immunometric methods.

**Results:**

The median urinary lithium concentration was 3,910 μg/L (5th, 95th percentiles, 270 μg/L, 10,400 μg/L). Median plasma concentrations (5th, 95th percentiles) of T_4_ and TSH were 17 pmol/L (13 pmol/L, 21 pmol/L) and 1.9 mIU/L, (0.68 mIU/L, 4.9 mIU/L), respectively. Urine lithium was inversely associated with T_4_ [β for a 1,000-μg/L increase = −0.19; 95% confidence interval (CI), −0.31 to −0.068; *p* = 0.002] and positively associated with TSH (β = 0.096; 95% CI, 0.033 to 0.16; *p* = 0.003). Both associations persisted after adjustment (for T_4_, β = −0.17; 95% CI, −0.32 to −0.015; *p* = 0.032; for TSH: β = 0.089; 95% CI, 0.024 to 0.15; *p* = 0.007). Urine selenium was positively associated with T_4_ (adjusted T_4_ for a 1 μg/L increase: β = 0.041; 95% CI, 0.012 to 0.071; *p* = 0.006).

**Conclusions:**

Exposure to lithium via drinking water and other environmental sources may affect thyroid function, consistent with known side effects of medical treatment with lithium. This stresses the need to screen for lithium in all drinking water sources.

We recently reported high concentrations of lithium, arsenic, cesium, rubidium, and boron in groundwater in the Puna region in the Argentinean Andes, probably related to influence of thermal water on the drinking water source (Concha et al. 2010). Similar lithium concentrations in drinking water (e.g., 1–3 mg/L) have been reported from northern Chile (Barr et al. 1993), but little is known about the prevalence and geographic distribution of lithium in drinking water in general. A study of trace elements in 132 brands of bottled water from 28 countries reported a median lithium concentration of 4.8 μg/L, with a total range of 0.06–5,460 μg/L (Krachler and Shotyk 2009), suggesting that the findings in Argentina and Chile are not unique. However, there appears to be neither drinking water standards nor risk assessments for lithium in drinking water.

The highest levels of lithium in drinking water in the Puna region were detected in the village of San Antonio de Los Cobres (1,005 μg/L; Concha et al. 2010), with little variation over the period measured (years 2004–2008). If we assume a stable exposure, the urinary lithium concentrations in women from San Antonio de los Cobres suggests a total daily lithium intake of 2–30 mg, including 2–3 mg from drinking water (Concha et al. 2010). For a woman weighing 50 kg, this level of exposure is within an order of magnitude of lithium doses used for maintenance treatment of bipolar disorder, 1–5 mg lithium/kg body weight/day (Grandjean and Aubry 2009a). Major side effects of long-term lithium therapy include thyroid abnormalities (presenting mainly as hypothyroidism and goiter), weight gain, edema, gastrointestinal pain, diarrhea, tremor, polyuria, and renal tubular damage (Aral and Vecchio-Sadus 2008; Grandjean and Aubry 2009b). Because of these well-recognized effects of lithium treatment, we wanted to evaluate potential effects of chronic exposure to lithium through drinking water and other environmental sources. We recruited women in a number of Puna villages in the Argentinean Andes with a wide range of lithium concentrations in drinking water supplies (8–1,005 μg/L; Concha et al. 2010) and evaluated the potential impact of urinary lithium on the concentrations of free thyroxine (T_4_) and the pituitary gland hormone thyrotropin [thyroid-stimulating hormone (TSH)] in plasma. We also evaluated associations between urinary lithium concentration and body mass index (BMI).

## Materials and Methods

### Study site and subjects

The Puna is the arid part of the Andean Mountains in northern Argentina, at an altitude around 3,000–4,000 m. Most study participants (*n* = 161) were from the village of San Antonio de los Cobres, province of Salta, with about 5,000 inhabitants, mainly of indigenous origin (Concha et al. 2010). Additional participants lived in the small Puna communities Olacapato (*n* = 14), Salar de Pocitos (*n* = 5), and Tolar Grande (*n* = 22), located 60–187 km southwest and west of San Antonio de los Cobres and each with < 200 inhabitants. In each village, all individuals had drinking water from the same source, with small variations in element concentrations over time (Concha et al. 2010).

Element concentrations in drinking water from different sources vary greatly within short geographic distances, mainly because of the presence of volcanic bedrock. Therefore, long-term exposure to lithium is difficult to assess for people not living most of the time in a defined area, where information about levels in drinking water is available. Thus, in the present study we focused on women because they spend more time close to home than do men, who may work outside the monitored region (e.g., in the city of Salta or in Chile) for long periods of time.

In San Antonio de los Cobres, women were recruited by physicians and community health workers at the local hospital. In the other villages, women were recruited with the assistance of personnel at local health centers or during visits to homes of village inhabitants. Although it was not possible, for practical reasons, to select the women at random, the community health workers at the hospital and health centers tried to recruit women from the whole district, to get as wide a geographic distribution of households as possible. There were no inclusion/exclusion criteria for study participation other than sex. No children < 12 years of age were recruited. The women were interviewed about what kind of water they consumed (tap water/bottled water and source of the tap water), their dietary habits (what do you eat for breakfast, lunch, and dinner?), diseases, and time of residence in the area, including that of their parents and grandparents. These questions revealed that all women almost exclusively drank tap water, their diets (mainly corn, beans, chicken, and pork) were very similar, and only 3 of 202 women reported ongoing use of any medication: One was being treated for gastritis and two for high blood pressure. Their time of residence in the area and other characteristics are presented in Table 1. Height and weight were measured using standardized protocols, and the women were asked to donate a urine sample (~ 20 mL) and blood samples (4 mL for separation of plasma). All the samples were collected at the hospital or the local health clinics during the daytime, and study participants were allowed to eat and drink before sampling. All the volunteers gave oral and written consent. The study was approved by the Ministry of Health in Salta, Argentina, and the ethical committee at Karolinska Institute (Sweden).

### Sample collection

Spot-urine samples were collected in disposable paper cups and immediately transferred to 20-mL acid-washed polyethylene bottles. After testing for pH and presence of protein and glucose by urine test strips (Siemens Multistix, Tarrytown, NY, USA), the urine samples were frozen at −20°C. Markers of thyroid function were measured in plasma, which was obtained from collected venous blood samples (heparin tubes) that were centrifuged within 10 min after blood withdrawal. Plasma and urine samples were kept at −20°C until they were transported (with cooling blocks) to Sweden for analysis.

### Analysis of elements and markers of thyroid function

We evaluated arsenic, boron, and cesium in urine because our previous study had demonstrated that these elements were elevated in drinking water in the same regions as lithium (Concha et al. 2010). Of note, cesium is known to accumulate in the thyroid (Bandazhevsky 2003). We also evaluated selenium and iodine because these elements are important for thyroid function [Lu and Holmgren 2009; World Health Organization (WHO) et al. 2007]. The urinary samples were analyzed 3 months after sampling using inductively coupled plasma mass spectrometry with collision/reaction cell (Agilent 7500ce; Agilent Technologies, Waldbronn, Germany) as described previously (Concha et al. 2010). Iodine was measured after diluting the urine with 0.1% NH_4_OH (Suprapur; Merck, Darmstadt, Germany). Analytical accuracy was ascertained by analyzing commercially available urine reference materials (Concha et al. 2010). For iodine, we analyzed Seronorm Urine No. 2525 (reference value, 282 μg/L) and OK4636 (analytical value, 139 μg/L; SERO AS, Billingstad, Norway) and obtained measured values of 294 ± 7.5 and 133 ± 4.4 μg/L (*n* = 11), respectively. To compensate for dilution, we measured specific gravity of urine using a digital refractometer (EUROMEX RD 712 clinical refractometer; EUROMEX, Arnhem, the Netherlands), and adjusted measured analyte concentrations to the mean specific gravity of all samples (1.020 g/mL). Free T_4_ and TSH were measured in 100 μL plasma by an immunometric method with reagents from Roche Diagnostics (Mannheim, Germany), performed as a routine analysis at the Department of Clinical Chemistry, Lund University. All plasma samples were stored for approximately 1 year at −20°C before analysis of T_4_ and TSH. According to the literature (El Ezzi et al. 2010; Männistö et al. 2007), the concentrations of T_4_ and TSH are very stable over time.

### Statistical analysis

We calculated the associations between characteristics of study subjects, urinary concentrations of selected elements, and plasma T_4_ and TSH levels using the Spearman correlation coefficient (*r*_s_). Associations between markers of thyroid function and BMI (dependent variables) and the selected elements, age, and parity (independent variables) were first analyzed with univariate linear regression analysis. Then multivariate models were used to estimate associations between urinary lithium concentration and T_4_, TSH, and BMI adjusted for other influential variables (defined as independent variables with *p*-values < 0.25 in the univariate linear regression analysis). Potential influential variables were the elements arsenic, boron, and cesium, which are elevated in drinking water in the same regions as lithium; and age, parity, and BMI because these factors may influence the markers of thyroid function. All statistical analyses were performed using IBM SPSS Statistics (version 18.0; SPSS Inc., Chicago, IL, USA). Associations were considered statistically significant based on an alpha level of 0.05.

## Results

Study participants had a median age of 34 years and parity of three children (Table 1). The median BMI was 24.7 kg/m^2^ (in the upper range of normal weight, 18.5–24.99 kg/m^2^), and 17% of the women (*n* = 35) were considered obese (> 30 kg/m^2^). T_4_ values were outside of the normal range (12–22 pmol/L) in 5 (2.6%) of 194 women (1 below and 4 above normal range), and TSH values were outside the normal range (0.4–4.0 mIE/L) in 20 (10%) of 196 women (3 below and 17 above normal range). Ninety-seven percent of the women reported that they had lived > 2 years in the area. Table 1 also shows the concentrations of toxic and essential elements in urine.

Lithium, boron, arsenic, and cesium in urine were all negatively correlated to free T_4_ (Table 1), but we found no significant correlations between these elements and TSH. Selenium, but not iodine, in urine was positively correlated to T_4_ and inversely correlated to TSH. Age, BMI, and parity were all negatively associated with T_4_ but not with TSH. We found a negative correlation between reported number of ingested glasses of water per day and TSH (*r*_s_ = −0.17; *p* = 0.015), but water consumption was not correlated with T_4_ (*p* = 0.36).

**Figure 1:**
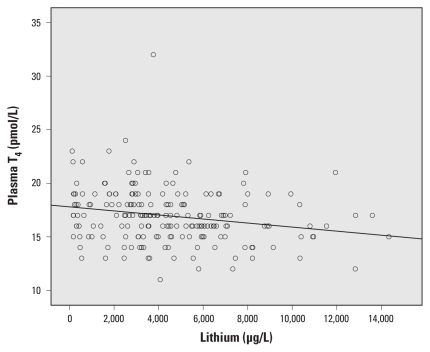
Association between urinary lithium concentration and plasma free T_4_.

**Figure 2:**
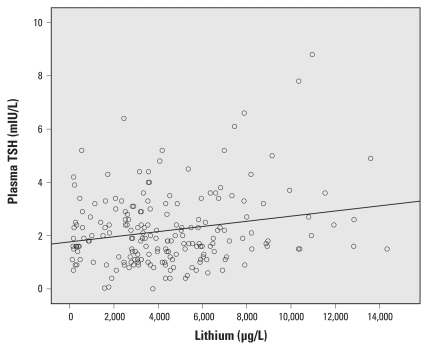
Association between urinary lithium concentration and plasma TSH.

Urine lithium concentration was inversely associated with free T_4_ before and after adjustment [Table 2, Figure 1; adjusted β for a 1,000-μg/L increase in urinary lithium concentration = 0.17; 95% confidence interval (CI), −0.32 to −0.015; *p* = 0.032] and positively associated with TSH (Table 2, Figure 2; adjusted β = 0.089; 95% CI, 0.024 to 0.15; *p* = 0.007). The effect of lithium on TSH was still present when we adjusted for arsenic, cesium, selenium, iodine, age, parity, and BMI (β = 0.14; 95% CI, 0.054 to 0.022; *p* = 0.001). For comparison, the β-coefficient point estimates for the associations between other covariates and markers of thyroid function and BMI are shown in Supplemental Material, Table 1 (doi:10.1289/ehp.1002678). Estimates were similar when we limited multivariate analyses to women who had lived > 2 years in the area (*n* = 179–180; for T_4_, β = −0.17; 95% CI, −0.32 to −0.011; *p* = 0.036; for TSH: β = 0.15; 95% CI, 0.063 to 0.23; *p* = 0.001). We found no association between lithium and T_4_ or TSH values outside the normal range (data not shown).

**Figure 3:**
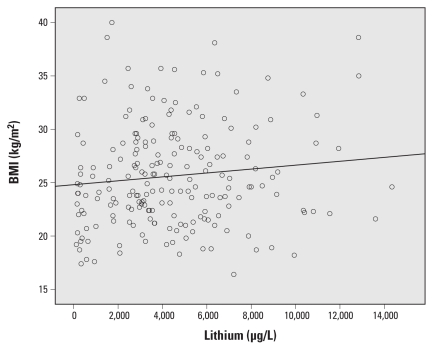
Association between urinary lithium concentration and BMI.

We found a nonsignificant positive association between urinary lithium and BMI (Table 2, Figure 3). The association between lithum and BMI became less significant after additional adjustment for T_4_ and TSH (β = 0.068; 95% CI, −0.015 to 0.28; *p* = 0.53).

In contrast with lithium, urinary selenium was positively associated with T_4_ (unadjusted β for a 1-μg/L increase in selenium = 0.05; 95% CI, 0.022 to 0.078; *p* = 0.001) and inversely associated with TSH (β = −0.014; 95% CI, −0.029 to 0.001; *p* = 0.069). However, only the association with T_4_ was statistically significant after adjustments [see Supplemental Material, Table 1 (doi:10.1289/ehp.1002678)]. Urinary boron was significantly negatively associated with T_4_ (β for a 1,000 μg/L increase = −0.068; 95% CI, −0.11 to −0.025; *p* = 0.002) but was not associated with TSH in univariate analyses. However, because of high collinearity (*r*_s_ = 0.85; *p* < 0.001) between boron and lithium, we did not include boron in multivariate analyses of associations between lithium and T_4_, TSH, or BMI. When boron was modeled instead of lithium in a multivariate analysis, we found no association between boron and TSH or BMI (*p* = 0.34 and *p* = 0.48) but it was inversely associated with T_4_ (β = −0.075; 95% CI, −0.14 to −0.013; *p* = 0.018). Cesium and arsenic were not associated with T_4_ or TSH (see Supplemental Material, Table 1).

## Discussion

This is to our knowledge the first study investigating the potential health impact of long-term lithium exposure from drinking water and other environmental sources on the thyroid. We found an association between lithium exposure, as assessed by lithium concentrations in urine, and markers of thyroid function. The positive associations between lithium and TSH and the inverse association between lithium and T_4_ are consistent with reports of hypothyroidism as a side effect of chronic use of lithium medication (Grandjean and Aubry 2009b; Lazarus 2009). Lithium treatment is also associated with weight gain (Grandjean and Aubry 2009b); however, we noted only a small, nonsignificant association between urinary lithium concentration and BMI in our study population. The positive association with T_4_ and inverse association with TSH suggest that selenium may have had a beneficial effect on thyroid function in this population, but, contrary to expectations, we did not observe similar associations with iodine. Cesium was not associated with T_4_ or TSH, despite accumulation of cesium in the thyroid (Bandazhevsky 2003).

The main strength of our study is the use of a biomarker of lithium exposure in urine combined with analysis of biomarkers for selenium, iodine, and cesium—all elements that may influence thyroid function—as well as sensitive markers in plasma of effect on the thyroid. However, the study has a few weaknesses. First, several elements were highly correlated in both drinking water and urine (Concha et al. 2010); thus, it is not possible to account for potential confounding by other elements, particularly boron. Nevertheless, whereas lithium was associated with both T_4_ and TSH, boron was associated with T_4_ only. To our knowledge, there are no data that show that boron is toxic to the thyroid; because little is known about the toxicology of boron, the possibility that boron may influence T_4_ cannot be excluded. In contrast, arsenic and cesium were not associated with TSH or T_4_. A second limitation is that we were not able to enroll a random sample of women, although we tried to recruit a representative population of the women living in this area. Finally, the cross-sectional nature of the study limits our ability to determine temporal relations between lithium exposures and the outcomes assessed. We believe that exposure levels would have been relatively stable over time, and that they would have begun very early in life. In support of this notion, the levels of lithium in drinking water, which contributes to a substantial fraction of the lithium in urine, have been stable during the period measured (2004–2008; Concha et al. 2010), and most of the women had lived in the area > 2 years.

Apart from our study, there are very few reports on the health effects of chronic lithium exposure besides the side effects of medication. In a recent study of suicide risk in relation to lithium concentrations in tap water in 18 municipalities of Oita prefecture in Japan, Ohgami et al. (2009) reported that the standardized mortality ratio for suicide was inversely associated with water lithium concentrations. In that study, water concentrations ranged from 0.7 to 59 μg/L, in contrast with a range from 8 to 1,005 μg/L in our study population (Concha et al. 2010). The Oita study has also been criticized for not considering lithium intake via food, which may reach > 100 μg/day (Desai and Chaturvedi 2009). In the present study, we estimated associations with urinary lithium concentrations, a biomarker of total exposure.

T_4_ was negatively correlated, and TSH weakly positively correlated with BMI. These associations may have been due to an effect of lithium on the thyroid, but we could not prove it statistically. In contrast with lithium, selenium was inversely associated with TSH and positively associated with T_4_. The possibility of an effect of selenium on thyroid function is consistently correlated with thyroid markers, which is in accordance with the proposed role of selenoenzymes, including deiodinase activities (Lu and Holmgren 2009). However, because the deiodinases regulate the conversion of T_4_ to the thyroid hormone triiodothyronine (T_3_), we would expect selenium to be inversely associated with T_4_. Selenium is also present in selenoproteins such as glutathione peroxidase and the thioredoxin reductase family, which are important for protection of the thyroid gland from excess hydrogen peroxide and reactive oxygen species produced by the follicles for biosynthesis of thyroid hormones (Kohrle and Gartner 2009; Lu and Holmgren 2009; Prummel et al. 2004). We found no association between urinary iodine and the thyroid markers among the women in our study population, most of whom had adequate iodine status (WHO et al. 2007), with 62% having urinary concentrations > 100 μg/L. This suggests that the observed associations of lithium with markers of thyroid function were not due to confounding by low iodine concentrations. Because the iodine concentrations in this population were relatively high, we could not analyze the combined effect of high lithium and low iodine concentrations on thyroid function.

Further studies on geographic distribution of elevated lithium concentrations in drinking water, as well as studies of the effects of exposure to lithium via drinking water and other environmental sources in women, men, and children, are highly warranted. The observed wide range of drinking water lithium concentrations among villages in the Puna region reinforces the need to screen all drinking water sources globally for many more potentially toxic elements than currently recommended.

## Figures and Tables

**Table 1:** Characteristics, measured concentrations of elements and hormones in urine and plasma, and correlations between the variables and T_4_ and TSH (*n* = 202).^a^

Variable	Mean	Median	5th, 95th percentiles	Minimum–maximum	Spearman rank (*r*_s_) correlation	
					T_4_	TSH
Age (years)	37.2	34	18, 64	12–80	−0.19**	−0.048
Years living in the area	24.7	25	3, 51	0.3–71	−0.096	−0.012
BMI (kg/m^2^)	25.6	24.7	18.8, 35	16–40	−0.24**	0.063
Parity (*n*)	4.3	3	0, 11	0–14	−0.28#	−0.064
Urinary concentration (μg/L)						
Lithium	4,404	3,914	268, 10,392	117–14,343	−0.25**	0.10
Boron	15,000	14,578	2,602, 27,907	1,292–45,313	−0.024**	0.025
Arsenic	244	230	21, 545	10–1,250	−0.20**	0.002
Cesium	473	470	26, 880	8.0–2,243	−0.27#	−0.005
Selenium	28	25	11, 51	4.0–74	0.23**	−0.18*
Iodine	147	125	37, 354	16–857	0.035	−0.063
Plasma concentration						
T_4_ (pmol/L)	17	17	13, 21	11–32	—	−0.11
TSH (mIU/L)	2.2	1.9	0.68, 4.9	0–8.8	−0.11	—

a All values were based on 202 observations with the exception of years living in the area (*n* = 198), parity (*n* = 197), plasma T_4_ (*n* = 194), and TSH (*n* = 196), because of either missing information (years living in the area and parity) or insufficient amount of sample left for analysis (plasma T_4_ and TSH).

* *p* < 0.05

** *p* < 0.01, and

# *p* < 0.001, for the Spearman’s correlations between the variables and T_4_ and TSH.

**Table 2:** Association between a 1,000-μg/L increase in urine lithium concentration and markers of thyroid function and BMI by linear regression analysis.^a^

Outcome	β-Coefficient	95% CI	*p*-Value
T_4_			
Unadjusted	−0.19	−0.31 to −0.068	0.002
Adjusted^b^	−0.17	−0.32 to −0.015	0.032
TSH			
Unadjusted	0.096	0.033 to 0.16	0.003
Adjusted^c^	0.089	0.024 to 0.15	0.007
BMI			
Unadjusted	0.18	−0.039 to 0.40	0.11
Adjusted^d^	0.14	−0.059 to 0.35	0.16

a Adjusted models include covariates with *p* < 0.25 in a univariate analysis, apart from boron, which was highly correlated to lithium (*r*_s_ = 0.85; *p* = 0.001).

b Adjusted for urinary arsenic, cesium, and selenium; age; parity; and BMI.

c Adjusted for selenium and BMI.

d Adjusted for urinary iodine, age, and parity.

## References

[b1-ehp-119-827] Aral H, Vecchio-Sadus A (2008). Toxicity of lithium to humans and the environment—a literature review. Ecotoxicol Environ Saf.

[b2-ehp-119-827] Bandazhevsky YI (2003). Chronic Cs-137 incorporation in children’s organs. Swiss Med Wkly.

[b3-ehp-119-827] Barr RD, Clarke WB, Clarke RM, Venturelli J, Norman GR, Downing RG (1993). Regulation of lithium and boron levels in normal human blood: environmental and genetic considerations. J Lab Clin Med.

[b4-ehp-119-827] Concha G, Broberg K, Grandér M, Cardozo A, Palm B, Vahter M (2010). High-level exposure to lithium, rubidium, cesium, boron and arsenic via drinking water in the Puna region of northern Argentina. Environ Sci Technol.

[b5-ehp-119-827] Desai G, Chaturvedi SK (2009). Lithium in drinking water and food, and risk of suicide. Br J Psychiatry.

[b6-ehp-119-827] El Ezzi AA, El-Saidi MA, Kuddus RH (2010). Long term stability of thyroid hormones and DNA in blood spots kept under varying storage conditions. Pediatr Int.

[b7-ehp-119-827] Grandjean EM, Aubry JM (2009a). Lithium: updated human knowledge using an evidence-based approach: part I: clinical efficacy in bipolar disorder. CNS Drugs.

[b8-ehp-119-827] Grandjean EM, Aubry JM (2009b). Lithium: updated human knowledge using an evidence-based approach: part III: clinical safety. CNS Drugs.

[b9-ehp-119-827] Kohrle J, Gartner R (2009). Selenium and thyroid. Best Pract Res Clin Endocrinol Metab.

[b10-ehp-119-827] Krachler M, Shotyk W (2009). Trace and ultratrace metals in bottled waters: survey of sources worldwide and comparison with refillable metal bottles. Sci Total Environ.

[b11-ehp-119-827] Lazarus JH (2009). Lithium and thyroid. Best Pract Res Endocrinol Metab.

[b12-ehp-119-827] Lu J, Holmgren A (2009). Selenoproteins. J Biol Chem.

[b13-ehp-119-827] Männistö T, Surcel HM, Bloigu A, Ruokonen A, Hartikainen AL, Järvelin MR (2007). The effect of freezing, thawing, and short- and long-term storage on serum thyrotropin, thyroid hormones, and thyroid autoantibodies: implications for analyzing samples stored in serum banks [Letter]. Clin Chem.

[b14-ehp-119-827] Ohgami H, Terao T, Shiotsuki I, Ishii N, Iwata N (2009). Lithium levels in drinking water and risk of suicide. Br J Psychiatry.

[b15-ehp-119-827] Prummel MF, Strieder T, Wiersinga WM (2004). The environment and autoimmune thyroid diseases. Eur J Endocrinol.

[b16-ehp-119-827] WHO (World Health Organization), UNICEF, and International Council for the Control of Iodine Deficiency Disorders (2007). Assessment of Iodine Deficiency Disorders and Monitoring Their Elimination: A Guide for Programme Managers.

